# Genetic Variations and Haplotype Diversity of the Wheat *FRIZZY PANICLE* (*WFZP*) Gene in 98 *Aegilops tauschii* Accessions

**DOI:** 10.3390/genes16040414

**Published:** 2025-03-31

**Authors:** Ruilong Tao, Shengke Li, Jia Liao, Fahui Ye, Shuxiang Yin, Jicheng Shen, Qingshan Cui, Xinfeng Wang, Deguo Song, Wenjie Chen, Shunzong Ning

**Affiliations:** 1 Laboratory for Research and Utilization of Qinghai Tibet Plateau Germplasm Resources, Academy of Agriculture and Forestry Sciences of Qinghai University (Qinghai Academy of Agriculture and Forestry Sciences), Xining 810016, China; 2 Triticeae Research Institute, Sichuan Agricultural University, Chengdu 611130, China; 3 Qinghai Provincial Key Laboratory of Crop Molecular Breeding, Northwest Institute of Plateau Biology, Chinese Academy of Sciences, Xining 810008, China; 4 University of Chinese Academy of Sciences, Beijing 100049, China

**Keywords:** wheat *FRIZZY PANICLE* (*WFZP*) gene, natural variation, spikelet, bread wheat, *Aegilops tauschii*

## Abstract

Background: The wheat *FRIZZY PANICLE* (*WFZP*) gene is a regulatory hub that controls spikelet formation in bread wheat, *WFZP-D*, as a favorable gene for wheat yield improvement. The sequences of *WFZP-D* in bread wheat accessions are known to be highly conserved. Methods: In this study, re-sequencing of *WFZP* homoeologous genes from 98 widely distributed *Aegilops tauschii* (the donor of the wheat D genome) germplasms was carried out to identify natural variations at both the nucleotide and polypeptide levels. Conclusions: *WFZP* homeolog exhibited high conservation with no functional variants in the key AP2/ERF domain. Haplotype characterization identified five haplotypes (Hap-D1 to Hap-D5) based on nine single-nucleotide polymorphisms, five of which induced single amino acid residue substitutions downstream of the AP2/ERF domain. Hap-D1 (identical to *Triticum aestivum WFZP-D*) and Hap-D2 are two most common. Hap-D1 is concentrated in Iran and Azerbaijan, primarily associated with ssp. *strangulata*, while Hap-D2 displays broad distribution across the range and primarily belongs to ssp. *tauschii*. The remaining haplotypes (Hap-D3/4/5) are identified in ssp. *tauschii* accessions. These findings suggest that strategic integration of ssp. *tauschii* into wheat-breeding programs could enhance genetic diversity. The identified natural variations provide potential haplotype resources for improving wheat yield potential.

## 1. Introduction

Bread wheat (*Triticum aestivum* L., AABBDD, 2n = 6x = 42) is a crucial cereal crop and provides about 40% of calories for the human population (http://faostat.fao.org, accessed on 5 May 2022). Enhancing wheat yield potential and stability is a priority for global food security. Grain yield is mainly determined by the number of grains produced in each inflorescence [1] and is a multi-factorial trait determined by multiple quantitative trait loci (QTLs). The number of fertile spikelets per spike is a determinant of the final grain number per spike at harvest and is determined early during reproductive development; it is also less affected by later environmental conditions [2] and has the highest heritability. Several key genes have been identified as regulators of spikelet number, such as the major genes of the flowering pathway [3], including *Photoperiod-1* (*Ppd-1*) and *FLOWERING LOCUS T* (*FT*), *LEAFY* (*LFY*) [4], *WHEAT ORTHOLOG OF APO1* (*WAPO1*) [4], and *Compositum2* (*COM2*) genes [5,6]. Among them, *Ppd-1* is a key regulator of inflorescence architecture and paired spikelet development in wheat by regulating the expression of the FT to increase spikelet number [6]. Insensitivity allele *Ppd-1a* decreases the number of spikelets per spike by shortening the pre-anthesis period [7,8,9]. FT mutations contribute to increased spikelet number but delay the heading date [10,11]. While Specific combinations of *LFY* and *WAPO1* natural alleles increase spikelet number per spike in wheat, loss-of-function mutations in *LFY* or its interacting protein WAPO1 significantly reduce the rate of formation of spikelet meristems [4]. The *Compositum2* (*COM2*) gene encodes the APETALA2/Ethylene Responsive Factor (AP2/ERF TF), which was identified in compositum (com) barley mutants with branched spikes and plays a crucial role in the determination of spikelet meristem fates and shoot meristem identity [5,6].

Recently, numerous major and stably expressed QTLs for supernumerary spikelets were also detected in the short arms of 2D chromosomes, especially those close to the wheat *COM2* orthologue (*WFZP)* genomic region [12,13,14]. *WFZP*, as a *COM2* orthologue in wheat-encoding class II AP2/ERF TF, has been characterized by the analysis of wheat multirow spikes (*mrs1* mutants) [15]. *WFZP* is a homoallelic series of loci in the short arms of the 2A, 2B, and 2D chromosomes of bread wheat: *WFZP-A*, *WFZP-B*, and *WFZP-D*, respectively. Simultaneous mutations in the coding regions of *WFZP-A* and *WFZP-D*, coupled with transposable element (TE) insertions in the *WFZP-B* promoter region, lead to a severe multirow spike [15]. Recently, it was confirmed that in the endemic Tibetan wheat variety Zang734, which exhibits typical multirow spikes with three spikelets per rachis node (defined as triple spikelets), the null mutation in *WFZP-A* and complete deletion of *WFZP-D* trigger triple spikelets [16]. Moreover, in *wfzp-d* single mutants, the spikelet/grain numbers per spike also significantly increased, but the spike architecture was completely normal. Furthermore, natural sequence variations were screened in 228 hexaploid wheat cultivars, and the *WFZP-D* locus exhibited no sequence variations in any of the wheat accessions, indicating an extremely low variation rate in wheat *WFZP-D* [16]. The transfer and harnessing of potential *WFZP-D* natural variations from donor species in wheat may be useful for genetic manipulations by increasing spikelets.

*Aegilops tauschii* (DD, 2n = 2x = 14) is the donor of the bread wheat D genome [17,18,19,20], as well as a pivotal genome in several *Aegilops* tetraploid and hexaploid species [21]. This species is adapted to a wide range of environments, and its distribution range stretches from West Turkey to East China [20,22]. The use of diverse accessions from different geographical origins as donors is important in identifying as many alleles or haplotypes as possible [23,24]. Synthetic hexaploid wheat (SHW) was produced by crossing *T. turgidum* with *Ae. tauschii*, and these plants exhibit many valuable agronomic characteristics, such as high grain weight and spikelet number or large kernels and spikes [25,26,27]. SHW is known to be an effective genetic resource for the utilization of natural variations from donor species to bread wheat [26,27]. Here, we describe the identification of the natural variation in donor species *Ae. tauschii* germplasms for the *WFZP* homeolog gene across diverse geographical distributions and to provide genetic resources for effective introduction and utilization in wheat yield improvement.

## 2. Materials and Methods

### 2.1. Plant Materials

The plant materials comprised randomly chosen 98 *Ae. tauschii* accessions that collectively encompassed a wide geographic distribution (Table S1). The materials were grown in the field at the Wenjiang Experimental Station of Sichuan Agricultural University in Chengdu, China. A distribution map of accessions was created based on the origin or geographic coordinates of the accessions, using the map_data package and the ggplot2 package for R (ver. 3.4) [28].

### 2.2. Primer Design, Plant DNA Amplification and Sequencing

Genomic DNA was extracted from freshly harvested leaves using a plant genomic DNA kit (Tiangen Biotech Co., Ltd., Beijing, China). Relevant PCR primers were designed based on the *WFZP* homeolog (AET2Gv20232900) in *Ae. tauschii* from the EnsemblPlants database (http://plants.ensembl.org/index.html (accessed on 27 March 2025)) by utilizing DNAMAN v6.0 (Lynnon Biosoft, Vaudreuil, QC, Canada) software. The *WFZP-D* sequence of *Ae. tauschii* was amplified using the primers WFZP-8F (5′-CTCCATAGTGAGCACTACC-3′) and WFZP-8R (5′-GCCTCTCGAGTACTCTCG-3′) (1478-bp amplicon). The amplicons were performed using a Veriti 96-Well Thermal Cycler (ABI, Waltham, MA, USA). Amplification of the DNA was carried out in 20 µL reactions containing 1 µL (10 µmol/L) of each primer, 1 µL (100 ng/µL) of template DNA, 10 µL of 2 × Taq Master Mix (Dye Plus) (Vazyme, Nanjing, China), and 7 µL double-distilled (dd) H_2_O. Each reaction was exposed to a denaturation step at 95 °C for 5 min, followed by 39 cycles of 95 °C/40 s, 61 °C/45 s, 72 °C/100 s, and a final extension at 72 °C for 10 min. The amplicons were electrophoresed on a 1.0% agarose gel in 1 × TAE buffer (0.04 mol/L Tris base, 0.02 mol/L acetic acid, and 1.0 mmol/L EDTA) and were visualized under UV light with ethidium bromide. The PCR products (1478 bp) were purified and sequenced directly using Sanger ABI 3730 sequencer (Applied Biosystems) by Qingke (Chengdu, China).

### 2.3. Protein 3D Modeling Prediction

To predict the protein 3D structure, we used AlphaFold v2.1, an open-source code [29]. We input the amino acid sequence into AlphaFold v2.1 and obtained five unrelaxed models, five relaxed models, and five ranked models in pdb format. Among these output models, the ranked_1.pdb model had the highest confidence, as indicated by the best Local Distance Difference Test (lDDT) score, and was therefore utilized. The structural graphics and the positions of amino acid substitutions were visualized using PyMOL (v. 2.6.0a0).

### 2.4. Sequence Alignments and Phylogenetic Study

Multiple sequence alignments at both the nucleotide and predicted polypeptide levels were conducted by DNAMAN v6.0 software (Lynnon Biosoft, Vaudreuil, QC, Canada). The *FZP* homologous sequences obtained from the National Center Biotechnology Information (https://www.ncbi.nlm.nih.gov/), including *WFZP-A* of *T. aestivum* (MH544619), *T. turgidum* (MH544637), *T. dicoccoides* (*XM037625360*), *T. urartu* (XM048704520) and *T. monococcum* (MH544636), *WFZP-B* of *T. aestivum* (MH544628) and *T. dicoccoides* (XM037626174), *Hordeum vulgare FZP* (XM045113517), *Lolium rigidum FZP* (XM047207198), Brachypodium distachyon *FZP* (XM003559789), *Oryza sativa FZP* (MN387799), *Sorghum bicolor FZP* (XM002463317), *Zea mays FZP* (XM008672421), *Arabidopsis thaliana FZP* (NM120840), and haplotypes of *WFZP-D* (including *T. aestivum*) were used to construct the phylogenetic trees. The neighbor-joining method in MEGA v11 software was used to construct the Phylogenetic trees [30], with bootstrap values based on 1000 replicates.

## 3. Results

### 3.1. Ae. tauschii WFZP-D: Nucleotide Sequence Polymorphism

**Figure 1:**
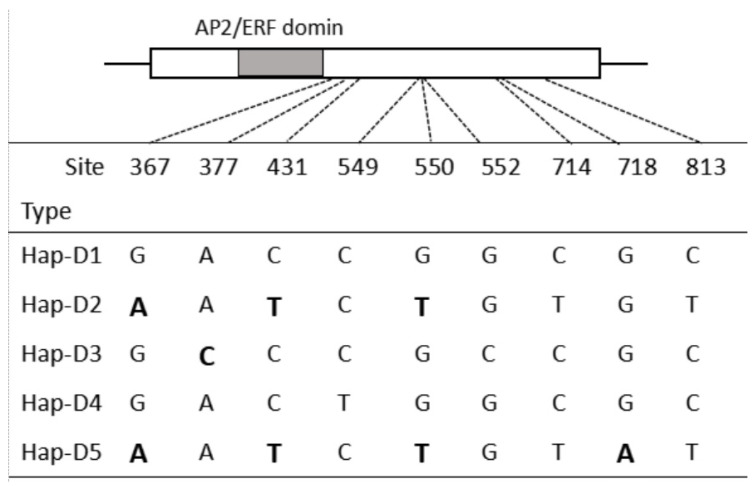
Haplotype variations in *WFZP-D* homeolog of D genome in the 98 accessions of *Ae. tauschii*. The grey box indicates the AP2/ERF domain. Exonic polymorphisms generating a changed peptide indicated in bold.

**Figure 2:**
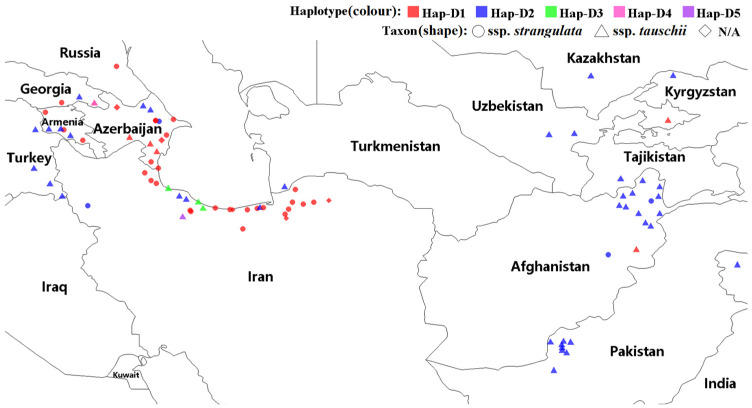
The distribution of the *Ae. tauschii* accessions used in the study and their allocation.

The highly conserved *WFZP* homoeologous sequences from 98 *Ae. tauschii* accessions were deposited in GenBank as accession PP907061 to PP907065 (Table S1). All homeologs had one exon according to the annotation of *T. aestivum WFZP-D* (MH544629) and the sequence of the coding region was 942 bp without variations. Nine single-nucleotide polymorphism (SNP) loci were detected, all of which were located outside of the AP2/ERF domain. Only five of these nine SNPs resulted in an altered peptide sequence (Figure 1). The 9 SNPs were used to divide the 98 homeologs into five haplotypes (Table S1, Figure 1 and Figure 2). Hap-D2, as the most common haplotype, was present in 49 accessions, the majority of which belonged to ssp. *tauschii*, except for five accessions. Hap-D2 was widely distributed among accessions from other countries, namely Afghanistan (13 accessions), Pakistan (8 accessions), Turkey (4 accessions), Iran (4 accessions), Armenia (3 accessions), Azerbaijan (3 accessions), and Uzbekistan (2 accessions). Hap-D1 was the same as the *T. aestivum WFZP-D* haplotype and existed in 44 accessions, the majority of which belonged to ssp. *strangulata* and were mainly distributed in Iran and Azerbaijan. Hap-D3 was present in three accessions and mainly located in Iran, while both Hap-D4 and Hap-D5 were present in only one accession. Hap-D3, Hap-D4, and Hap-D5 belonged to ssp. *tauschii*.

### 3.2. Ae. tauschii WFZP-D: Peptide Sequences

**Figure 3:**
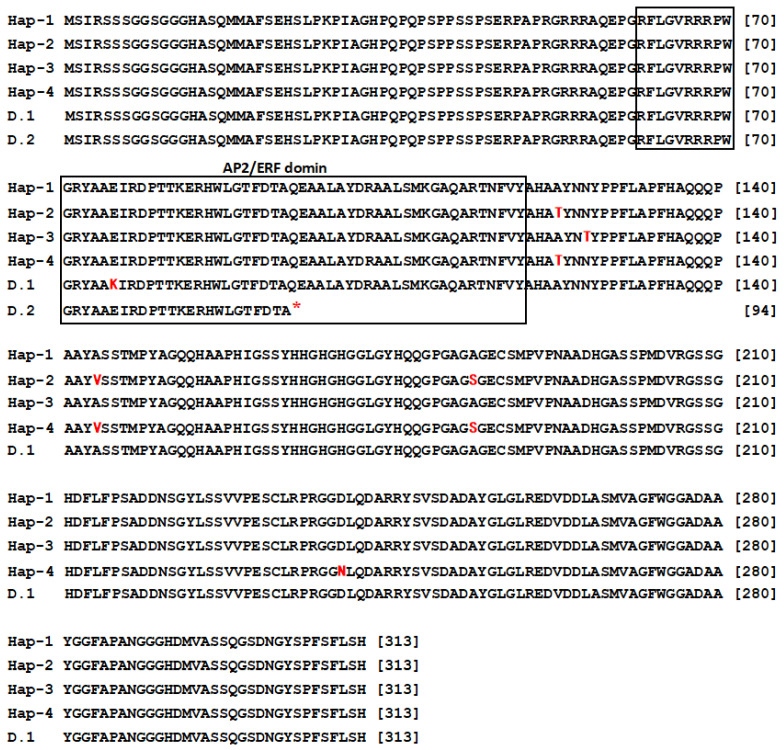
Alignment of the polypeptide sequences of WFZP-D homeolog. Polymorphisms generating a changed peptide compared with Hap-1 (*T. aestivum WFZP-D* haplotype) are indicated in red. The key feature of the AP2/ERF domain is shown in boxes. D.1 (MH544630) and D.2 (MH576978) are the sequences of the wfzp-D.1 (multirow spike) and wfzp-D.2 (supernumerary spikelet) mutations, respectively.

At the polypeptide sequence level, the five haplotypes from *Ae. tauschii* were classified into four distinct polypeptides. Polypeptide alignment of *WFZP* homeolog proteins is shown in Figure 3. The predicted proteins from the nucleotide sequences of Hap-D1 and Hap-D4 had the same polypeptide and were termed the Hap-1 polypeptide haplotype, while the predicted proteins of the nucleotide sequences of Hap-D2, Hap-D3, and Hap-D5 were named Hap-2, Hap-3, and Hap-4, respectively. The predicted protein of *WFZP-D* (MH544629) in bread wheat was indistinguishable from that of Hap-1. The E to K mutation (wfzp-D.1) and premature termination codon mutation (wfzp-D.2) in the AP2/ERF domain (Figure 3) caused supernumerary spikelet and multirow spike phenotypes in wheat, respectively [15]. All WFZP homeologs shared conserved key features of AP2/ERF proteins (boxed in Figure 3). Five altered amino acids were found downstream of the AP2/ERF domain. One altered amino acid was different from that of bread wheat (Hap-1) in Hap-3, an N to T change occurred at position 126. Three altered amino acids were from A to T at position 123, from A to V at position 144, and from A to S at position 184 in Hap-2 and Hap-4. The remaining one altered amino acids was from D to N at position 238 in Hap-4 (Figure 3).

**Figure 4:**
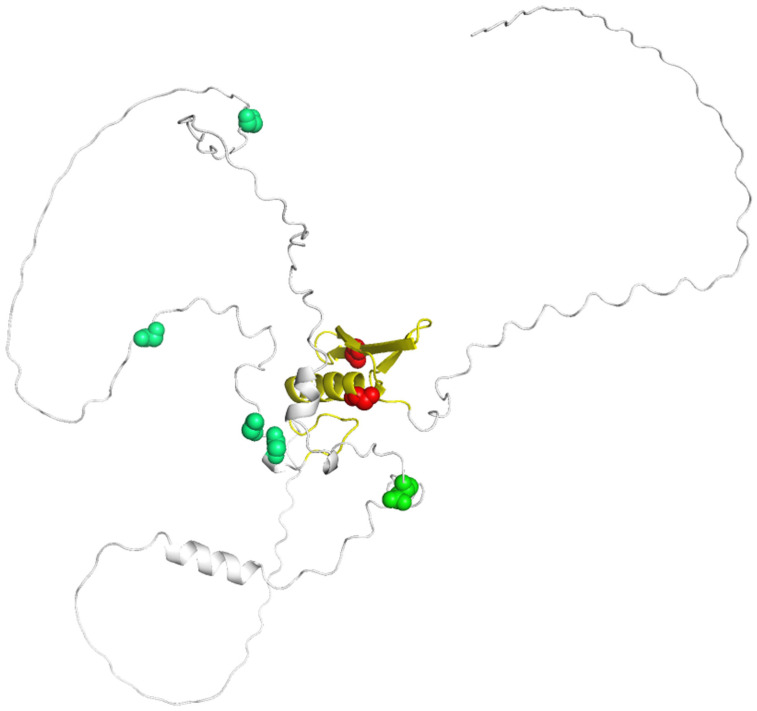
Protein structure prediction. Polymorphisms resulting in a changed peptide compared with Hap-1 are marked in green. The wfzp-D.1 (multirow spike) and wfzp-D.2 (supernumerary spikelet) mutations are marked in red; AP2/ERF domain is marked in golden yellow.

To predict the 3D structure of WFZP-D, a detailed analysis revealed that mutations were observed in specific regions of the AP2/ERF domain (Figure 4). Specifically, in WFZP-D.1, a mutation occurred in the β-sheet region, while in WFZP-D.2, the mutation was found in the α-helix region. These mutations within the key structural domains of the protein suggest potential alterations in its functional properties. Additionally, mutations were identified in the Hap-1, Hap-2, Hap-3, and Hap-4 regions at distinct positions within the protein sequence; however, these mutations did not significantly affect the overall conformation of the protein.

### 3.3. Phylogeny of the WFZP Homoeologs

**Figure 5:**
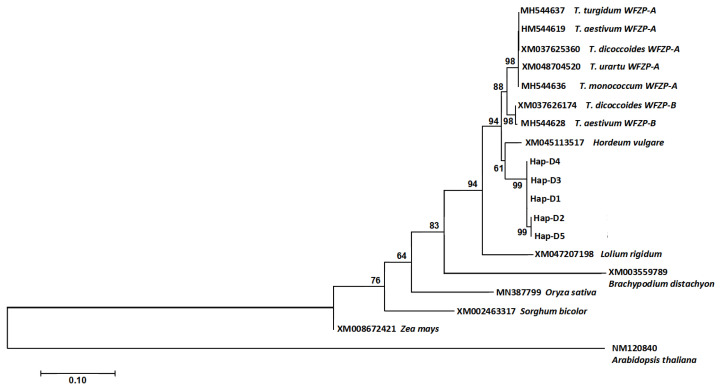
Phylogeny based on the full-length genomic sequence of *A. thaliana*, *B. distachyon*, *L. rigidum*, *S. bicolor*, *O. sativa*, *Z. mays*, *H. vulgare*, bread wheat, tetraploid wheat, diploid wheat, and *Ae. tauschii WFZP* homeologs using the neighbor-joining method. The *FZP* homologous sequence of *Arabidopsis thaliana* (NM120840) was used as the outgroup. Only bootstrap values (%) >50% are shown.

Phylogeny based on nucleotide sequences of wheat *WFZP* homeolog genes (Figure 5) revealed that each of the three homeologs from *Triticum*, *Ae. tauschii*, and barley were divided into three clades. The groups from the A and B genome were more closely related to each another than to the D genome and barley group. Among the homeologs from the D genome group, two subgroups were recognizable: one clustered the sequences Hap-D1 (including *T. aestivum*), Hap-D3, and Hap-D4, and the other clustered the sequences of *Ae. tauschii* Hap-D2 and Hap-D5. The homologous sequence of cereal, including *Ae. tauschii*, *Triticum*, *Hordeum*, *Lolium*, *Brachypodium*, *Oryza*, *Sorghum*, and *Zea*, clustered into one large clade and showed that they were phylogenetically closely related to one another. The *FZP* homologous sequence of *Arabidopsis thaliana* (NM120840) was used as the outgroup.

## 4. Discussion

Increasing spikelet number could be an effective strategy for increasing wheat yield [31,32,33]. To date, several crucial genes that regulate the spikelet number in wheat have been reported. For example, *Ppd-1* is a member of the pseudoresponse regulator (*PRR*) gene family that controls photoperiod sensing and heading date in wheat. It acts as a key regulator of inflorescence architecture and spikelet formation by modulating the expression of *FT* and other inflorescence determination genes [3]. Mutations in the insensitivity allele (*Ppd-1a*) are associated with a photoperiod-insensitive phenotype [34,35]. *Ppd-1a* decreases the number of spikelets per spike [7,9,35,36,37,38,39,40] by shortening the floret initiation phase [8,41,42]. Furthermore, *Ppd-1a* in common wheat enables it to adapt to different environmental changes [37], corresponding to variations in photoperiod response adapted to different environments [43]. Although the photoperiod-sensitive phenotype *ppd* is a potential means to increase wheat yield potential, it is difficult to utilize this phenotype for effective breeding. While *FT* mutations have been shown to contribute to increased spikelet number, they also delay the heading date [10,11]. The allele combination of *LFY* and *WAPO1* can be used in wheat breeding, but specific combinations need to be identified and then assembled [4]. As a regulative hub for controlling spikelet formation in bread wheat, *WFZP* is a favorable gene for yield improvement [15,16]. *WFZP* represses the expression of the spikelet-formation gene *TaBA1* by acting on its promoter [16]. It has been confirmed that the null mutation in *WFZP-A* and complete deletion of *WFZP-D* triggered triple spikelets in the endemic Tibetan wheat variety Zang734, which exhibits triple spikelet per rachis node [16]. Surprisingly, *wfzp-d* single mutants exhibited increased spikelet/grain numbers per spike and completely normal spike architecture [16]. Moreover, no sequence variations were detected in wheat *WFZP-D* in 228 hexaploid wheat cultivars [16]. This finding suggests that exploring natural variations in *WFZP-D* is a potential means for achieving high yield by increasing spikelets.

The *Ae. tauschii* population harbors rich haplotypic diversity. Structural analysis revealed that *WFZP* homoeologous sequences from *Ae. tauschii* accessions contain a single exon, with sequence variations predominantly localized outside the AP2/ERF domain (Figure 1). We identified five haplotypes based on nine SNPs, four of which induced single amino acid substitutions (Figure 1, Figure 3 and Figure 4). Notably, mutations within conserved AP2/ERF domains (e.g., maize *bd1* and rice *FZP*) are known to trigger premature stop codons or nonfunctional proteins, altering inflorescence architecture [44,45]. A 4-bp deletion in the 5′-regulatory region of *FZP* decreased its transcriptional activity and significantly increased the number of secondary branches and grain yield in rice [46]. Furthermore, *WFZP* exhibits dual roles to promote nitrogen utilization, through modulating root systems and the expressions of N-related genes [47]. Therefore, the potential value of a newly discovered altered peptide sequence from *Ae. tauschii* is worth evaluating in the future.

Previous studies have revealed that *WFZP-A/WFZP-D* double mutants display compromised agronomic performance compared to single mutations (*WFZP-A* or *WFZP-D*), indicating a gene dosage-dependent regulatory mechanism of WFZP in maintaining optimal developmental traits [16]. Building on these findings, we propose a future research strategy involving the knockout of *WFZP-A* coupled with the overexpression of *WFZP-D* to evaluate whether this genetic manipulation further enhances agronomic traits. This dual approach offers significant potential for elucidating the role of *WFZP* in regulating spike development and related traits in wheat. Consistent with prior evidence linking *Ae. tauschii* ssp. *strangulata* to the D-genome origin of bread wheat [48,49,50,51], our analysis revealed complete congruence between Hap-D1 and the *T. aestivum WFZP-D* haplotype. This congruence was observed in 44 accessions predominantly classified as ssp. *strangulata* (Figure 2 and Table S1). Notably, newly identified haplotypes including Hap-D2 have not been found in common wheat varieties, the vast majority of which belonged to ssp. *tauschii,* except for five accessions. Hap-D3, Hap-D4, and Hap-D5 also belonged to ssp. *tauschii*. Furthermore, geographic analysis of 90 accessions with verified origin data (Figure 2 and Table S1) demonstrated exclusive distribution of Hap-D1 in Iran and Azerbaijan. To elucidate the tripartite relationship between *WFZP-D* gene variants, *Ae. tauschii* subspecies, and their biogeography, future investigations should prioritize expanded sampling with precise geographic metadata.

## 5. Conclusions

The wheat *FRIZZY PANICLE* (*WFZP*) gene serves as a regulatory hub controlling spikelet formation in bread wheat. More importantly, *WFZP-D* has been identified as a favorable gene for wheat yield improvement. Analysis of *WFZP-D* was conducted across 98 widely distributed accessions of *Ae. tauschii*, donor of the wheat D genome. This analysis revealed the presence of five haplotypes, distinguished by nine single-nucleotide polymorphisms (SNPs), with four of these SNPs resulting in single amino acid substitutions outside the AP2/ERF domain. Notably, *WFZP* homeolog in *Ae. tauschii* are highly conserved. The distribution of these haplotypes is noteworthy: Most accessions carrying Hap-D1 (which shares the same sequence as *T. aestivum WFZP-D*) are concentrated in a specific region, whereas the majority of accessions with Hap-D2 are widely distributed from Turkey to Kyrgyzstan. These Hap-D2 accessions primarily belong to ssp. *tauschii,* with only five exceptions. Furthermore, Hap-D2, the most prevalent haplotype, contains five single-nucleotides variations. The effective utilization of the genetic resources present in ssp. *tauschii*, which exhibit high sequence variation, are very important for transferring these genes to widely cultivated wheat species.

## Data Availability

The original data presented in the study are openly available in NCBI GenBank (https://www.ncbi.nlm.nih.gov/) under accession numbers PP907061 to PP907065.

## Supplementary Materials

The following supporting information can be downloaded at: https://www.mdpi.com/article/10.3390/genes16040414/s1, Table S1: Source of germplasm utilized.
